# A green tea extract catechin EGCg: Therapeutic potential for pediatric cardiomyopathies

**DOI:** 10.1002/pdi3.7

**Published:** 2023-06-09

**Authors:** Junjun Quan, Dustin Gerber, Ang Li, Xupei Huang, Jie Tian

**Affiliations:** ^1^ Department of Anesthesiology Children’s Hospital of Chongqing Medical University National Clinical Research Center for Child Health and Disorders Ministry of Education Key Laboratory of Child Development and Disorders Chongqing Key Laboratory of Pediatrics Chongqing China; ^2^ Department of Cardiology Children's Hospital of Chongqing Medical University Chongqing China; ^3^ Department of Biomedical Science Charles E. Schmidt College of Medicine Florida Atlantic University Boca Raton Florida USA

**Keywords:** (−)‐epigallocatechin‐3‐gallate, cardiomyopathy, diastolic dysfunction, green tea, heart failure

## Abstract

Cardiomyopathies comprise a group of disorders wherein the primary defect is in cardiac myocytes. The common forms of pediatric cardiomyopathies, classified according to their morphological and functional manifestations, include dilated cardiomyopathy (DCM), restrictive cardiomyopathy (RCM), hypertrophic cardiomyopathy (HCM), and others. Cardiac gene mutations caused abnormal myofibril Ca^2+^ sensitivity may be involved in the underlying molecular mechanisms of cardiomyopathies. Thus far, no effective treatment for cardiomyopathies has been developed, especially for HCM and RCM in which diastolic dysfunction occurs early and followed by diastolic heart failure. Our laboratory is among the first in the field to investigate the mechanisms underlying various cardiomyopathies and search for the treatment for these disorders. In the past, we and other researchers have found that (−)‐epigallocatechin‐3‐gallate (EGCg), the major biomedical polyphenol extracted from green tea, possess multiple therapeutic effects on protecting cardiac function and correcting impaired relaxation. Given its therapeutic effects, EGCg might be a potential drug candidate for administration to patients with cardiomyopathy and heart failure. In this review, we will discuss the molecular mechanisms associated with the pathogenesis of diastolic dysfunction and summarize the pharmacological effects of EGCg on experimental animals and pediatric patients with cardiomyopathies and diastolic dysfunction.

## 1 INTRODUCTION

Cardiomyopathy (CM) is a common heart disease in children that leads to cardiac dysfunction. Among three major types of cardiomyopathies, dilated cardiomyopathy (DCM), hypertrophic cardiomyopathy (HCM), and restrictive cardiomyopathy (RCM), HCM and RCM share a common pathological feature, that is, diastolic dysfunction and diastolic heart failure (DHF), whereas the main manifestation in DCM is systolic dysfunction.^1^ The symptom of pediatric CM depends on the presence of heart failure (HF), especially on the type of CM and severity.^2^, ^3^ The pathologic and clinical features of pediatric CM are shown in Table 1.^4^, ^5^, ^6^

**TABLE 1:** The pathologic and clinical features of pediatric cardiomyopathies.

Type	Cardiac structure and functions	Symptoms	Endomyocardial biopsy
RCM	Restrictive filling and reduced diastolic volume of either or both ventricles Diastolic dysfunction Normal or near‐normal systolic function	Older children: Shortness of breath and fatigue, dizziness or lightheadedness, syncope, persistent cough, and palpitations Newborns and babies: Difficulty feeding, poor growth, excessive sweating with feeds or activity, excessive fussiness, or dyspnea	Moderate interstitial fibrosis Cellular hypertrophy without myocardial infiltrative or storage disease or endocardial pathology
HCM	The increasing severity of left ventricular hypertrophy Left ventricular outflow tract obstruction Diastolic dysfunction Normal or near‐normal systolic function	Cardiac arrhythmias, syncope, sudden cardiac death, embolic events	Hypertrophy of myocytes Marked interstitial fibrosis with hypertrophy and disarray of myocardial cells
DCM	Ventricular chamber enlargement Systolic dysfunction	Vomiting, diarrhea, poor feeding, failure to thrive, dyspnea, cardiogenic shock	Hypertrophy of myocytes Attenuation of the myocytes Endocardial smooth muscle cell hyperplasia

Abbreviations: DCM, dilated cardiomyopathy; HCM, hypertrophic cardiomyopathy; RCM, restrictive cardiomyopathy.

HF is a global public health crisis, including HF with reduced ejection fraction (HFrEF) and HF with preserved ejection fraction (HFpEF), which affects one's quality of life and increases the burden of mortality and morbidity.^7^ In recent decades, the incidence of HFpEF has been elevated, along with stable overall HF incidence and high risk for mortality.^8^ Compared to patients with HFrEF, patients with HFpEF always exhibit normal systolic function. HFpEF is also known as DHF, and the therapy remains difficult and is often ineffective because the mechanisms involved in diastolic dysfunction have not been interpreted clearly.^9^ Several laboratories, including ours, implicate myofilament protein mutations, abnormal sensitivity of myofibrils to Ca^2+^, and cardiac troponin I (cTnI) deficiency are likely as contributors and possible targets for compromised diastolic function,^10^, ^11^ which may become a therapeutic target for these abnormalities. (−)‐Epigallo‐Catechin‐3‐Gallate (EGCg), derived from green tea, has been recently reported to have various biological effects on the heart and can improve diastolic function and reverse impaired relaxation both in animal and clinical trials.^10^, ^12^

Traditionally, researchers pay more attention to Ca^2+^ concentration inside of myocardial cells since Ca^2+^ is a trigger that facilitates the cross‐bridge formation when Ca^2+^ binds with troponin. Several medications have been developed to improve cardiac contractility by increasing Ca^2+^ concentration in myocardial cells, that is, positive inotropic medications such as digoxin and β‐agonists. Recently, several studies have demonstrated that cardiac myofibril sensitivity to Ca^2+^ is important as well in the regulation of cardiac function especially the diastolic function.^10^ In the past 15 years, our laboratory has carried out the studies to investigate the relationship between Ca^2+^ hypersensitivity caused by myofibril protein mutations and the consequential diastolic dysfunction in the heart in transgenic mouse models.^10^ Furthermore, we have confirmed that desensitization, including a transgenic molecule with Ca^2+^ hyposensitivity and green tea extract catechin, is a useful tool to correct diastolic dysfunction caused by Ca^2+^ hypersensitivity in CM.^13^ Besides, the same process and therapeutic value have been observed in HCM mouse models.^14^ Experiments in vivo have shown that green tea extract catechin, calcium desensitizer, can reduce myofilament Ca^2+^ hypersensitivity via interacting with cardiac troponin‐C (cTnC).^15^ Green tea extract catechin has been proved to be a small molecule that targets on troponin and manifests a therapeutic potential of enhancement of cardiac diastole.^10^, ^14^, ^15^

**FIGURE 1:**
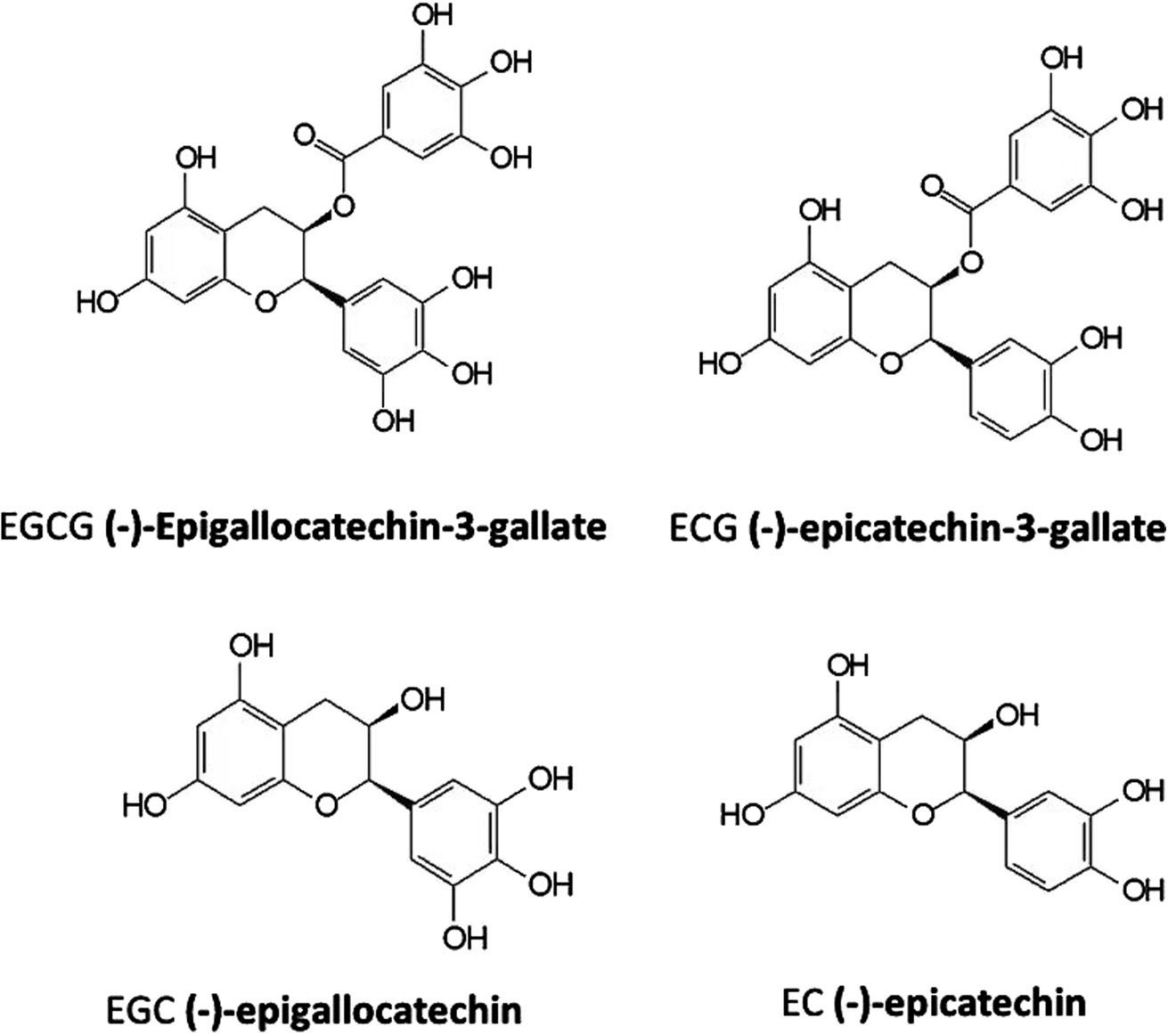
This figure presents the chemical structure of green tea catechins.

Green tea, obtained from the tea plant *Camellia sinensis*, is the most widely consumed beverage except for water. It originates from China and has spanned across numerous countries over thousands of years. Green tea is produced by the method of steaming the fresh leaves at a high temperature to deactivate the polyphenol oxidases, thereby preventing the oxidation of catechins and sustaining the monomeric forms of polyphenols. The predominant and medically relevant active components of green tea are polyphenols, which account for approximately 40% in dry weight, followed by protein content, lignin, amino acids, caffeine, organic acid, and chlorophyll. Among four main types of green tea extracts, EGCg is the major polyphenol ingredient (over 60%), followed by (−)‐epigallocatechin (EGC), (−)‐epicatechin (EC), and (−)‐epicatechin‐3‐gallate (ECG)^16^ (Figure 1). In the recent years, green tea catechins have been confirmed to possess potent calcium desensitization and anti‐cardiovascular, antioxidant, antidiabetic, antiallergic, and anticancer activity,^15^, ^17^, ^18^, ^19^ which may protect many organs for its wide range of biological activities. Cardiovascular diseases are responsible for more than 50% of the global mortality^20^; interestingly, several studies have shown that regular green tea consumption is inversely associated with their mortality.^17^, ^21^ This also suggests that green tea extract has a great application prospective in cardiovascular diseases, which is consistent with the experimental and clinical application in diastolic dysfunction and cardiomyopathies. In this review, we will address an update on recently reported molecular mechanisms involved in the development of diastolic dysfunction and the possible molecular mechanisms and pharmacological effects of EGCg, and summarize experimental and clinical application of green tea extract in pediatric CM and diastolic dysfunction.

## 2 EXPERIMENTAL TREATMENT USING EGCG IN ANIMALS WITH CM

### 2.1 EGCg improves ventricular compliance and reduces the internal muscle rigidity in RCM mice

Increased myofibril Ca^2+^ sensitivity with cTnI C‐terminal mutations is a key factor for leading to impaired relaxation and diastolic dysfunction as reported previously, suggesting myofibril Ca^2+^ hypersensitivity may be a potential therapeutic option. In vivo, we have demonstrated that Ca^2+^ desensitization, produced by introducing a hyposensitivity protein that can improve diastolic function and survival rate, is effective in rescuing the RCM mice with diastolic dysfunction.^13^ But it is difficult to apply such transgenic technology for human patients with diastolic dysfunction. Therefore, it is urgent to find Ca^2+^ desensitizers that can be used to reverse myofibril hypersensitivity to Ca^2+^ and impaired relaxation. And the biological agents should possess a good bioavailability with nontoxicity and can be used in live animals and human.

Recent studies have indicated that green tea extract EGCg, with a great bioavailability and safety, has a Ca^2+^ desensitizing ability via its interaction with cTnC.^15^, ^22^ The study in vivo revealed that after administration with 50 mg/kg EGCg for three months, the diastolic dysfunction parameters such as left ventricular end diastolic dimension (LVEDD) and isovolumetric relaxation time (IVRT) in RCM mice with cTnI R193H mutation were partially or completely returned to normal compared to the wild‐type mice, along with no significant changes in left ventricular wall thickness and systolic function. The results suggested that diastolic dysfunction could be corrected in RCM mice after 3‐month EGCg treatment and Ca^2+^ desensitizer EGCg was helpful to reverse the impaired relaxation caused by Ca^2+^ hypersensitivity in cTnI R193H RCM mice.^10^ In vitro, the result of acute 5 μM EGCg treatment significantly accelerating relaxation time and correcting delayed Ca^2+^ decay in RCM myocardial cells was similar to that in vivo, which indicated that EGCg could affect RCM myocardial cells directly via reducing Ca^2+^ hypersensitivity and improving impaired relaxation with no significant adverse effects.^10^ Meanwhile, there were no significant changes in cardiac electrical activities observed using ECG after the treatment with a high dose of EGCg, illustrating EGCg is safe to be used. Our other study showed that LVEDD was increased, and the prolonged IVRT was dramatically shortened in RCM mice after 3‐month treatment with EGCg, which was consistent with our previous report.^23^ In this study, we found EGCg was effective in correcting diastolic dysfunction in RCM mice mainly by improving ventricular compliance and reducing the internal muscle rigidity caused by myofibril hypersensitivity to Ca^2+^.^23^ And EGCg treatment did not regulate the levels of Ca^2+^ handling proteins such as and their phosphorylation status.^23^

The increase in the Ca^2+^ sensitivity, caused by high Ca^2+^ binding‐affinity, was also observed in RCM cardiomyocytes with cTnC G34S variant.^24^ EGCG showed the beneficial effects concerning thin filament integrity in vitro, probably via direct binding to actin and Ca^2+^ sensitivity of thin filament activation.^24^ However, green tea extract catechin so far has not been tested on RCM animals or human with cTnC variants.

### 2.2 EGCg accelerates Ca^2+^ dissociation from cTnC and restores the coupled relationship between Ca^2+^ sensitivity and troponin I phosphorylation in HCM mice

Currently, several studies have shown that elevated myofibril Ca^2+^ sensitivity induced by mutations in the N‐ or C‐terminal regions of troponin I plays a critical role in the development of HCM.^25^, ^26^ And the maximal Ca^2+^ ATPase activity can be regulated by cTnI phosphorylation. Specially, phosphorylation of the sites S23 and S24 can accelerate Ca^2+^ dissociation from cTnC to promote heart enter into rapid relaxation period^27^, ^28^ which is helpful to maintain proper Ca^2+^ sensitivity and cardiac function. Based on these roles of cTnI mutations, Ca^2+^ desensitizer EGCg is tried to treat the impaired relaxation in HCM. Other studies also showed that EGCg could alter Ca^2+^ sensitivity in thin filaments containing HCM‐causing cTnI mutations in a phosphorylation dependent manner and recover the coupled relationship between Ca^2+^ sensitivity and troponin I phosphorylation to normal in mutant thin filaments.^29^, ^30^

From the same therapeutic option for reducing Ca^2+^ sensitivity, green tea extract EGCg was also used in HCM with diastolic dysfunction caused by cardiac troponin T (cTnT) variants. A recent study showed that EGCg treatment could fully restore motility to the levels of wild‐type Ca^2+^ sensitivity and phosphorylation dependence in HCM transgenic mice with cTnT R92Q mutation.^31^ An in vitro study revealed that EGCg was a useful agent to treat the inherited HCM caused by increased myofilament Ca^2+^ sensitivity.^32^ The results demonstrated that 30 μM EGCg restored the impaired cardiac pump function due to diastolic dysfunction by reversing the increased Ca^2+^ sensitivity of cardiac myofilaments in △E160 cTnT HCM transgenic mice. Moreover, in vitro, EGCg was also confirmed to have an ability to reduce myofibril Ca^2+^ sensitivity, as exhibited by rightward shifts of the force‐pCa relationships with a significant decline in pCa50 and increasing the binding of the N‐terminal H1 helix region of cTnI to cTnC.^32^

The other laboratory also illustrated that the myofilament Ca^2+^ sensitivity was increased in HCM with cTnC mutation.^33^ The related cTnC mutations exerted their impacts mainly through changing the structural dynamics of the cTnC regulatory *N*‐domain and appeared to be antagonistic toward the effect of phosphorylation signaling from cTnI to cTnC.^34^ Although the myofilament Ca^2+^ sensitivity is observed in HCM caused by cTnC mutations, calcium desensitizer so far has not been administrated in this kind of HCM.

An HCM mouse model with an E180G mutation in α‐tropomyosin (Tm) demonstrated myofibril Ca^2+^ hypersensitivity, severe hypertrophy, and diastolic dysfunction,^35^ which was similar to the pathogenesis of HCM with troponin mutation. In this study, the double transgenic mice were crossed by Tm180 transgenic mice with mice with myofibril Ca^2+^ hyposensitivity caused by pseudophosphorylated cardiac troponin I (S23D and S24D; TnI‐PP). And the double transgenic mice illustrated no changes in the expression of phospholamban and sarcoplasmic reticulum Ca^2+^ ATPase, the increased levels of phospholamban and troponin T phosphorylation, and reduced phosphorylation of troponin I, indicating that desensitization of myofilament sensitivity to Ca^2+^ and associated correction of abnormal relaxation and diastolic dysfunction could delay or prevent the development of HCM and could be considered as a therapeutic target for HCM.^35^ However, it is difficult to apply such transgenic technology for human patients to reverse impaired relaxation and diastolic dysfunction. EGCg may be a great candidate compound to apply in animals and human who have HCM Tm mutations.

Recently, another thick filament protein, the myosin‐binding protein (*MYBPC3*) attracts more attention for its role in cardiac function and its mutation‐associated HCM.^36^ The mutation in *MYBPC3* has been found to cause HCM manifesting as left ventricular hypertrophy, hypersensitivity to Ca^2+^ and diastolic dysfunction. These studies supported demonstrated that the HCM phenotype could be reversed by decreasing myofilament Ca^2+^ sensitivity, indicating that targeting thin filament by decreasing Ca^2+^ sensitivity has a therapeutic importance.^35^, ^37^ However, whether this is also the target of EGCg on HCM with thick filament mutations is not known. In vitro studies have shown that1.8 µM EGCg could increase diastolic Ca^2+^, accelerate Ca^2+^ transient kinetics, and shorten relaxation time in isolated *MYBPC3‐*associated HCM myocardial cells.^14^ In addition, EGCg reduced myofibril Ca^2+^ sensitivity in *MYBPC3*‐targeted knock in skinned ventricular trabeculae.^14^ HCM cat hearts with *MYBPC3* R820W variant had a higher Ca^2+^‐sensitivity than non‐HCM cat hearts, and Ca^2+^ hypersensitivity in these HCM cats could be reversed by EGCg.^38^

All these studies suggest that myofibril Ca^2+^ hypersensitivity due to the mutations in cardiac thin or thick filaments play an important role in the development of HCM or RCM, which can be reversed by green tea catechin EGCg. It is noteworthy that EGCg, in vitro, at high dose of 50 μM increased the incidence of diastolic dysfunction via β2‐adrenoceptor, though EGCg at low dose of 20 μM conferred cardio‐protection,^39^ indicating that there may be a need for more studies to confirm the side effects and recommend the dose of EGCg in different kinds of breeds.

### 2.3 EGCg recovers the uncoupling of Ca^2+^ sensitivity and troponin I phosphorylation in DCM mice

**FIGURE 2:**
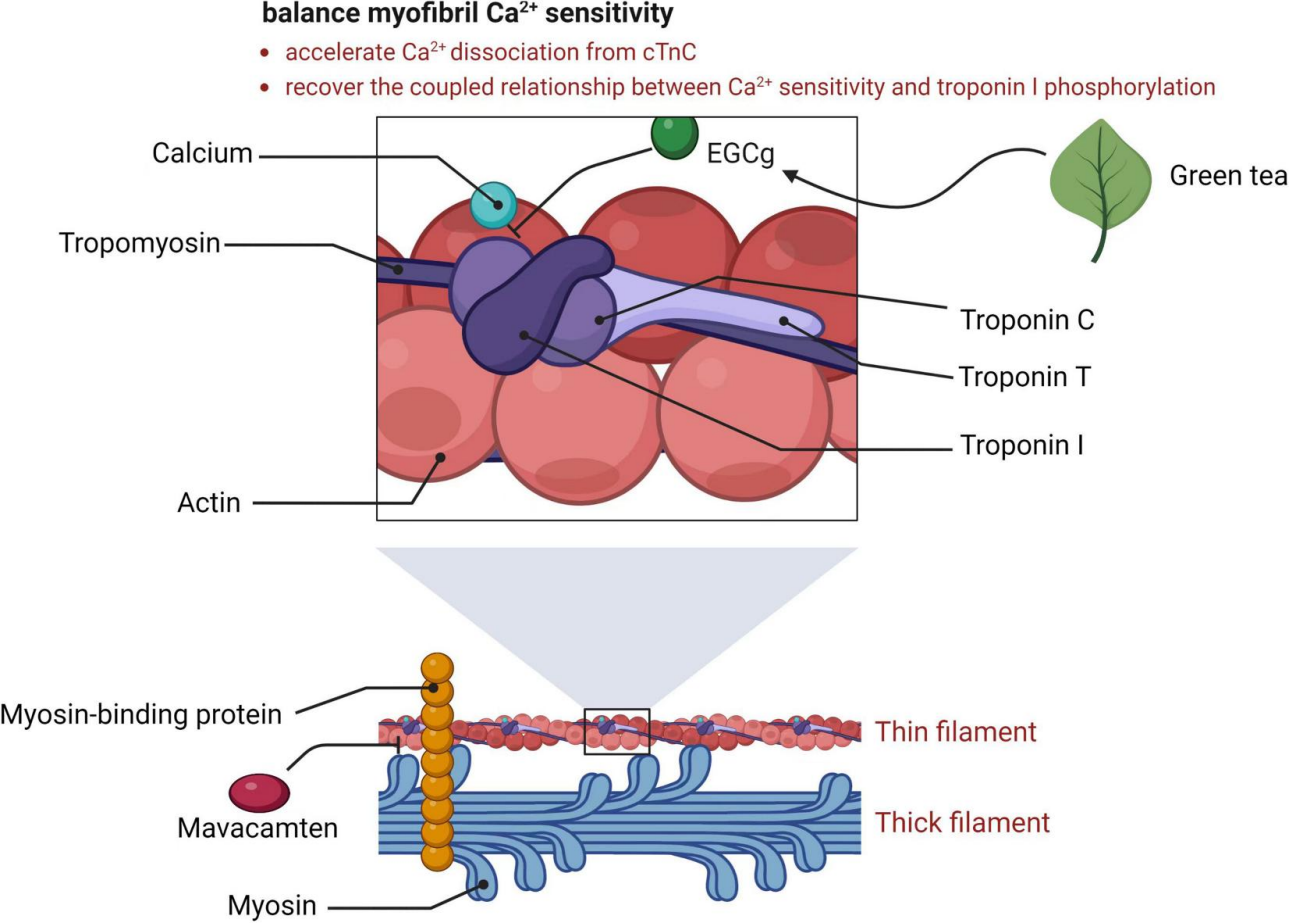
This figure summarizes the currently understood mode of actions of EGCg in cardiomyopathy. Mutations in cardiac proteins troponin and tropomyosin, myosin and myosin‐binding protein are the causes of cardiomyopathy. They are the targets of green tea extract EGCg as well. The therapeutic mechanisms of EGCG, focused on balancing myofibril Ca^2+^ sensitivity or recovering the coupled relationship between Ca^2+^ sensitivity and troponin I phosphorylation, for cardiomyopathy and diastolic dysfunction are similar. In contrast, the mechanism of mavacamten is to reduce actin‐myosin cross‐bridge formation.

Although DCM has different pathophysiological and morphological features compared with RCM and HCM, characterized with enlarged ventricle and systolic dysfunction, its causes are often from single mutations in a protein of the sarcomere. DCM is a complicated phenomenon. DCM‐related mutations are associated with a reduced Ca^2+^ sensitivity,^40^, ^41^ but numerous surveys now show that DCM‐related mutations in thin filament proteins can increase or decrease Ca^2+^ sensitivity.^42^, ^43^ However, the DCM phenotype is always linked to the loss of modulation of Ca^2+^ sensitivity by TnI phosphorylation (uncoupling), and this unstable relationship proposes causative of this disease with actin mutation.^29^ Interestingly, although myofibril Ca^2+^ sensitivity in DCM is much more complicated and green tea extract EGCg has been used in diastolic dysfunction, several studies have indicated that Ca^2+^ desensitizer EGCg is also called a re‐coupler and possesses the ability of restoring the coupled relationship between Ca^2+^ sensitivity and TnI phosphorylation in mutant thin filaments to normal,^29^, ^30^ which may be contributed to explore the treatments for DCM‐associated heart failure. Further studies are needed to confirm the positive effects of EGCg on rescuing the pathogenic process of DCM. Figure 2 presented a summary of molecular targets of EGCg in CM, created in BioRender.com.

### 2.4 EGCg is as experimental application in other cardiomyopathies with cardiac dysfunction

#### 2.4.1 Diabetic cardiomyopathy

Diabetic cardiomyopathy (DbCM) is present in almost two‐thirds of patients with type 2 diabetes mellitus, and hyperglycemia is a common risk factor for the development of HF.^44^, ^45^ Hyperglycemia‐induced metabolic alterations can facilitate cardiac dysfunction characterized by diastolic dysfunction followed by altered systolic function at later stages of the disease, resulting in symptomatic HF.^46^, ^47^ The available studies show that EGCg can suppress the pathophysiological processes involved in the progress of DCM, thereby improving heart function through various pathways.^48^, ^49^

#### 2.4.2 Doxorubicin‐induced cardiomyopathy

Doxorubicin is one of the effective antineoplastic agents to date for the widest scope of activity in human cancers. However, the drug effectiveness is severely limited by its cardiac side effects related to CM and heart failure.^50^, ^51^ Recent reports have suggested that EGCg could protect myocytes against oxidative stress‐induced cellular injury in doxorubicin‐treated cardiac myocytes, which may be applied to doxorubicin‐induced CM and related heart failure^50^, ^51^

### 2.5 EGCg improves cardiac functions of other cardiovascular diseases

#### 2.5.1 Age related cardiac diastolic dysfunction

The incidence of age‐related changes in diastolic function have been elevated in the elderly population.^7^, ^52^ Numerous studies have revealed that diastolic dysfunction increases with advancing age, and approximately half of the five million heart failure patients in the United States have been diagnosed with HFpEF. More than 90% of HFpEF patients are over the age of 60 at the time of diagnosis,^53^, ^54^, ^55^ suggesting that age per se is a major risk factor of diastolic dysfunction.

cTnI is critical in the regulation of cardiac function, especially diastolic function and its deficiency and mutations are associated with diastolic dysfunction and HEpEF.^11^, ^56^ The data from using a small samples of human heart tissues revealed that the concentration of cTnI in left ventricular myocardial cells decreased in the elderly with or without cardiac disease.^57^ Very recently, our research has shown that cTnI decrease in aging hearts might be one of the reasons that caused diastolic dysfunction of aged mice.^11^, ^58^ Green tea catechin EGCg not only has an effect on altered myofilament Ca^2+^ sensitivity, but it also regulates gene expression via epigenetic modifications. Our experimental results demonstrated that age‐related cardiac diastolic dysfunction caused by cTnI low expression was reversed by EGCg treatment through up‐regulating acetylated lysine 9 on histone H3 in aging hearts.^11^ The other data from our group indicated that EGCg administration could prevent heart failure via histone acetylation modifications as well.^59^

#### 2.5.2 Myocardial infarction

Myocardial infarction (MI) is a common cause of HFpEF, and emerging evidences indicates that impaired myocardial perfusion and inflammation secondary to multiple comorbidities are key mechanisms of HFpEF.^60^ In isolated rat hearts with 30 min of regional ischemia and two hours of reperfusion, 10 μM EGCG significantly improved ventricular function variables, including left ventricular developed pressure, rate‐pressure produce, maximum positive left ventricular pressure derivative (+d*P*/d*t*_max_), and minimum negative left ventricular pressure derivative (−d*P*/d*t*_min_) by using an MP150 pressure transducer.^61^ In an experimental heart failure model, green tea extract ameliorated E wave deceleration time, +d*P*/d*t*_max_, and −d*P*/d*t*_min_, suggesting green tea extract induces an improvement of systolic function and diastolic function after MI.^62^ And oxidative stress, energy metabolism, apoptosis, and extracellular matrix alterations may be potential mechanisms. Interestingly, oral pretreatment with EGCg could preserve cardiac function after ischemia‐reperfusion, an effect that may involve its antioxidative, antiapoptotic properties.^63^ In addition, a recent study, using a mouse model of heart failure, exhibited EGCg that had a function of attenuating myocardial injury and inhibiting the progression and development of heart failure.^64^ And this preventive effect of EGCg is likely mediated through the inhibition of myocardial fibrosis and reduction of ventricular collagen remodeling by lowering the transforming growth factor‐β1/mothers against the decapentaplegic homolog 3 signaling pathway.^64^

## 3 CLINICAL APPLICATION OF EGCG IN PATIENTS WITH HEART FAILURE AND CM

Heart failure is the leading cause of mortality in the United States.^65^ In two major types of HF, HFrEF, and HFpEF, the latter is nearly exclusively found in persons who are with old age or CM.^2^, ^66^, ^67^ The prevalence of HFpEF is rising with morbidity, mortality, and healthcare costs are now equal to HFrEF.^2^, ^7^, ^68^, ^69^, ^70^ Outcomes following hospitalization for decompensated HFpEF are poor, with about 1/3 of patients re‐hospitalized or dead within 90 days of discharge.^71^ Its pathophysiology is poorly understood, and no medication trials have had a positive effect on their primary endpoints. A major cause of HFpEF is diastolic dysfunction which is very common in older adults and pediatric patients with CM. Diastolic dysfunction refers to an impaired relaxation and an abnormality in heart filling during diastole while left ventricular systolic function is preserved.^35^, ^72^ There is a critical need to understand the mechanisms that cause diastolic dysfunction in an aging heart and develop target‐based medications.

As we discussed previously, myofilament Ca^2+^ sensitivity is one of the most critical factors for diastolic dysfunction. It is necessary to find Ca^2+^ desensitizers that primarily affect myofilament dynamics. So far, compounds with such properties are very scarce. Myosin inhibitors such as blebbistatin and 2, 3‐butanedione monoxime (BDM) may alter myofilament sensitivity to Ca^2+^ via their inhibitory effect on actomyosin cross‐bridge formations. These myosin ATPase inhibitors, while useful in functional studies in vitro and ex vivo, are too toxic for therapeutic use.^73^, ^74^

There is a great need to develop or find small molecules and chemical Ca^2+^ desensitizers that can be used to alter myofibril sensitivity for Ca^2+^ and myofilament sliding kinetics. Green tea extract catechin, EGCg, possess the Ca^2+^ desensitizing abilities and the potential to improve diastolic dysfunction as described previously.^15^, ^22^ EGCg is the most bio‐active compound in green tea and has numerous health benefits.^75^ EGCg desensitizes myofilament sensitivity to Ca^2+^ by forming a ternary complex with the C‐terminal domain of troponin C and the anchoring region of cTnI.^15^ Thus, the affinity of TnC to Ca^2+^ is reduced and cardiac relaxation is improved. The ability of EGCg to correct myofilament Ca^2+^ hypersensitivity and diastolic dysfunction has also been confirmed in HCM mouse models.^32^

Since patients with HFpEF are often highly symptomatic, and have a poor quality of life, medications used for it are generally to relieve symptoms and improve well‐being. Clinically, diuretics are usually used to reduce congestion to improve symptoms and signs of HFpEF. However, there is lack of evidences that anti‐HF drugs, β‐blockers and mineralocorticoid receptor antagonists could improve symptoms in these patients. And evidence of whether angiotensin receptor blocker and angiotensin‐converting enzyme inhibitors could have an ability of improvement in symptoms is inconsistent.^76^, ^77^, ^78^ These patients usually have a poor prognosis as a result and no treatments have been shown to reduce morbidity or mortality in HFpEF.^79^, ^80^

### 3.1 EGCg could improve diastolic dysfunction of pediatric patients with CM

In October 2006, EGCg was approved as a clinical medicine by the FDA, and several studies have indicated that EGCg has the potential of improving diastolic dysfunction. To confirm the effectiveness of green tea extract catechin, a clinical study was carried out in pediatric RCM and HCM patients with diastolic dysfunction. In this single‐center study, 12 subjects, age ranges from 0.8 to 14.2 years, were treated daily with a commercially available EGCg for 12 months. During the study, three patients that died of sudden death and heart failure were terminated from the study. The data obtained from this study have demonstrated that a daily intake of EGCg as a supplementary has beneficial effects in the tested subjects.^12^ The tested patients showed a significant improvement in cardiac function after the administration of EGCg, showing changes of heart failure levels from Class III to II or from Class IV to III. Meanwhile. A significant increase of left ventricular end diastolic volume and stroke volume and a significant decrease of IVRT were observed by echocardiography in the patients.^12^ These results were in accordance with our data in RCM mice with EGCg treatment. Interestingly, RCM patients with a mutation of sarcomere mutations in troponin I had a better response toward the treatment with EGCg. Similar to our study, the other group had also reported that the application of EGCg in pediatric RCM patients with cTnI mutations was effective by improving diastolic function and reversing the clinical symptoms in the patients.^81^ And there needs more trials and patients to enroll more patients with HFpEF to confirm the therapeutic effects of EGCg on heart failure.

Moreover, mavacamten is a novel, first‐in‐class, allosteric inhibitor of cardiac myosin ATPase, which reduces actin‐myosin cross‐bridge formation, thereby reducing myocardial contractility and improving myocardial energetic consumption in experimental HCM models. An adult clinical study (over 18 years old) to evaluate mavacamten (MYK‐461) approved by FDA in April 2022, with symptomatic obstructive HCM (EXPLORER‐HCM) started in 2018 with an enrollment of over 250 patients. The study reported that mavacamten was superior to placebo in reducing left ventricular outflow tract obstruction, improving exercise capacity, and ameliorating exercise capacity, New York Heart Association functional class and health status in patients with obstructive HCM.^82^, ^83^, ^84^ However, the use of mavacamten for pediatric patients with HCM is currently restricted due to a lack of data on the relationship of age to the effects of mavacamten in the pediatric population. Compared with mavacamten, EGCg with good drug safety is more advantageous in the treatment of pediatric CM.

### 3.2 EGCg is administrated in wild‐type transthyretin amyloid cardiomyopathy patients

Previous studies have reported the prevalence of wild‐type transthyretin amyloid cardiomyopathy (wtATTR‐CM) increases with age and that prevalence is estimated to be approximately 10%–25% in patients over 80.^54^, ^85^, ^86^ A Spanish study comprising patients with diastolic dysfunction demonstrated wtTTR amyloidosis in 13% of these patients.^86^ So far, wtTTR amyloidosis has been considered another important but under‐recognized contributor to age‐related diastolic dysfunction. Recently, in vitro experiments have shown that EGCg could inhibit fibril formation of diverse amyloidogenic proteins.^87^, ^88^, ^89^ And disruption of TTR fibrils was observed after a daily oral administration of 100 mg/kg EGCG for six weeks by using a transgenic mouse model of familial amyloidotic polyneuropathy carrying the human amyloidogenic Val30Met TTR variant.^90^ After a daily consumption of green tea for 12 months, a significant decline of left ventricular myocardial mass in patients with wtATTR‐CM was observed by cardiac magnetic resonance imaging, while no changes of left ventricular wall thickness or ratio of *E*/*E*’, scarce evidences to diagnose diastolic dysfunction were detected by echocardiography.^91^, ^92^ Thus, there still needs more data to confirm the effects of EGCg on age related wtATTR‐CM with diastolic dysfunction.

Except for the reports on CM patients, there are only a few studies about clinical trials of EGCg on cardiovascular diseases, though some clinical projects have revealed green tea extract can prevent colorectal adenomas, obesity, and other pathological states.

## 4 CONCLUSIONS

The prevalence of HFpEF continues to increase, and it accounts for significant morbidity and mortality. Diastolic dysfunction is a precondition associated with decreased active ventricular relaxation in early diastole and increased myocardial passive stiffness in late diastole. Several cardiovascular diseases including RCM, HCM, wtATTR‐CM, DbCM, and MI can merge HFpEF via different pathways. And molecular mechanisms in diastolic dysfunction may involve in the abnormal myofibril Ca^2+^ sensitivity, cTnI deficiency, unbalanced hemodynamics and metabolism. However, there are other mechanisms that still remain under exploration and need further studies. Therefore, more research of the potential mechanisms relating to diastolic dysfunction is needed to investigate novel cardioprotective strategies. Recently, polyphenols derived from green tea have been the focus of a number of studies due to their beneficial effects on health. The current available studies have demonstrated the protective effects of EGCG against the pathophysiological processes involved in the progress of diastolic dysfunction and HFpEF through regulation of myofibril Ca^2+^ sensitivity, elevation of cTnI expression, inhibition of fibril formation of amyloidogenic proteins and myocardial fibrosis, and reduction of ventricular collagen remodeling, suppression of oxidative stress damage etc. However, the clinical studies regarding EGCg applications in human patients are still limited. Therefore, further research on the pharmacological values of EGCg needs to be extended to the clinical arena to decrease the morbidity and mortality associated with diastolic dysfunction.

## AUTHOR CONTRIBUTIONS

Junjun Quan, Dustin Gerber, and Ang Li wrote the manuscript. Xupei Huang and Jie Tian initiated the idea, guided the article structure, and reviewed the final manuscript. All authors read and approved the final manuscript.

## CONFLICT OF INTEREST STATEMENT

Xupei Huang and Jie Tian are the members of the *Pediatric Discovery* Editorial Board. To minimize bias, they were excluded from all editorial decision‐making related to the acceptance of this article for publication. The remaining authors declare no conflict of interest.

## ETHICS STATEMENT

Not applicable.

## Data Availability

The data that support the findings of this study are openly available in NCBI at https://www.ncbi.nlm.nih.gov/.
